# FERTILITY CARE IN LOW- AND MIDDLE-INCOME COUNTRIES: Training in assisted reproduction in South Africa

**DOI:** 10.1530/RAF-24-0086

**Published:** 2025-10-08

**Authors:** Lenore Manderson, Andrea Whittaker, Trudie Gerrits

**Affiliations:** ^1^ University of the Witwatersrand Johannesburg – School of Public Health, Johannesburg, Gauteng, South Africa; ^2^ Monash University – Social Sciences, Melbourne, Victoria, Australia; ^3^ University of Amsterdam Faculty of Social and Behavioural Sciences – Anthropology, Amsterdam, Netherlands

**Keywords:** training, assisted reproduction, access to infertility treatment, South Africa

## Abstract

**Abstract:**

Intending parents on the African continent have limited access to quality services for infertility treatment. South Africa is the primary provider of fertility care on the continent, but because specialist training is only available in three public (teaching) hospitals, supported through partnerships with private institutions, there is a shortage of medical staff and waiting times for admission to training programmes can be years. We draw on data from our qualitative research study on assisted reproduction, generated from clinic visits, informal interviews, participation in science meetings, and formal interviews with 117 patients, gamete donors, clinicians, reproductive scientists and others to explore access to and motivation for training. Trainees’ reasons for embarking on this specialisation included: concern with limited access to gynaecological and fertility care on the continent; lack of skilled fertility specialists and embryologists; and lack of options for assisted reproduction available to low-income intending parents. Trainee fellows expressed commitment to low-cost IVF models to address the lack of affordable and accessible reproductive health care. Fertility specialists often shared this concern and emphasised the need for trained professionals to expand services. In general, interviewees felt that infertility care and assisted reproduction were regarded as of lesser importance to other reproductive and health problems, despite the extent of infertility and the demand for assisted reproduction technology (ART) on the continent.

**Lay summary:**

Countries across the African continent have the highest infertility rate in the world, yet access to diagnosis of cause, treatment and assisted reproductive technology is sparse. Assisted reproduction clinics now operate in several countries, particularly Ghana, Nigeria, Kenya and South Africa. Most support is in the private health system, and few women and men have access to low-cost assisted reproduction services at public hospitals. While clinics, biobanking services and laboratories are sparse, so too is training. We draw on data from a large study on assisted reproduction in South Africa to explore the provision of training. Most training is provided in South Africa, and people from other countries can access this. However, few teaching hospitals provide training, and people face long delays, sometimes years, before they can enrol. The limited opportunities for training seriously impact the capacity of countries to meet the health needs and support the reproductive hopes of many of their populations.

## Background

Sub-Saharan Africa (SSA) has the highest rate of infertility globally. Women and men routinely experience infections and other unresolved health conditions, which impact on women’s capacity to conceive, maintain pregnancy and deliver a healthy infant. Many countries struggle to provide comprehensive reproductive health care – competing health priorities and implementation challenges abound for economic, political and ideological reasons (Afferri *et al.* 2024), and many women lack access to services to address conditions that compromise conception. Competing health needs, social stigma associated with childlessness, and a lack of appropriate, available and affordable services consequently have a major impact on people living with infertility. Moreover, clinics are geographically clustered: South Africa (23 centres), Ghana (18 clinics), Kenya (11 clinics) and Nigeria (96 clinics) currently have the highest concentrations of clinics in SSA (Allan *et al.* 2019, Dyer *et al.* 2020, Horton *et al.* 2022, Boshoff *et al.* 2025). An estimated 1,500 assisted reproduction cycles per million infertile people are required to meet present need in SSA; in 2020 only 87 cycles per million took place (Afferri *et al.* 2022*a*). The absolute shortage and uneven distribution of *in-vitro* fertilisation (IVF) clinics result in poor access to quality services for those who need them (Whittaker *et al.* 2024).

In many countries, medical training in infertility management is limited, specialised assisted reproduction services may be unavailable, and only interventions such as ovulation induction, intrauterine insemination (IUI) and tubal surgery may be offered (Bittaye *et al.* 2023). One difficulty in expanding assisted reproduction services is the lack of clinical and laboratory staff who have been trained in and have expertise and experience in assisted reproduction and embryology (Afferri *et al.* 2022*b*); there are constant challenges to training and retaining staff due to ‘brain drains’ to facilities in other countries. Clinics therefore employ expatriates, often on a fly-in fly-out (FIFO) basis (Whittaker & Gerrits 2025), to fulfil the need for experienced embryologists, resulting in difficulties in setting up and running clinics. The transfer of costs to intending parents means that for many, ART is simply unaffordable (Adageba *et al.* 2015, Ombelet & Goossens 2016).

The lack of standardisation in training and clinical practice in assisted reproduction globally (de Ziegler *et al.* 2015*a*) also affects the quality of services, highlighting country-to-country disparities and differences in clinical practice, academic traditions and regulations overseeing training. Global capacity to meet the classical ‘by-fellowship’ training models has reduced at the same time as increased demand for reproductive endocrinology, infertility and assisted reproductive technologies specialists in LMIC. These issues have been identified by ANARA – the African Network and Registry for Assisted Reproductive Technology – as priorities, and the network has emphasised the importance of specialist training and continuing education. In this article, we provide a case study of REI-ART training in South Africa, including on the infrastructures of care as perceived by those involved in ARTs. We reflect upon the current system of training for REI-ART in South Africa in terms of the current opportunities for training, difficulties in the current system, professional socialisation of specialists, their motivation to specialize in this area, and their suggestions for improvement.

### Context of assisted reproductive treatment within South Africa

Within South Africa, access to ART is dependent upon a patient’s ability to pay (Whittaker *et al.* 2024). ART treatment in South Africa primarily occurs in the private sector in clinics and laboratories run by independent specialists. Coverage for ART is offered in a top-tier policy at the time of writing by two health insurers – discovery and CAMAF (Chartered Accountants Medical AID Fund) – with ongoing negotiations with other insurers. At the same time, less than 16% of South Africans, mostly white, have medical insurance (Statista - https://www.statista.com/statistics/1412884/number-of-medical-aid-beneficiaries-in-south-africa-by-population-group/), and poorer patients must rely on state-subsidised ART at overburdened public reproductive medicine units and face structural constraints within the public healthcare sector, including long waiting times and limited resources (Mabweazara 2024). Few public academic clinics offer means-tested subsidised IVF cycles (Botha *et al.* 2018): two in Cape Town and one each in Pretoria and Bloemfontein; the public academic clinic in Johannesburg is still being established. Even in public clinics, patients pay substantial out-of-pocket expenses, often resulting in debt (Dyer & Patel 2012, Dyer *et al.* 2013, 2017, Botha *et al*. 2018).

Medically assisted reproduction and the use of assisted reproductive technology are governed by the National Clinical Guidelines for Safe Conception and Infertility, published by the South African National Department of Health in 2020; surrogacy is covered by the Children’s Act 38 (2005). The guidelines for assisted reproduction state that healthcare providers at relevant clinics and day hospitals should receive ‘adequate training’, and that infertility care should be co-ordinated and mainly provided by a gynaecologist, with infertility specialist assistance, as provided, for instance, by an embryologist when assisted reproductive technology is required. New draft regulations circulated in 2021 cover newer technologies to allow non-invasive prenatal testing (NIPT) for chromosomal abnormality, and genome editing using CRISPRCas9 (Thaldar & Shozi 2022); these provisions remain under discussion.

The Southern African Society of Reproductive Medicine and Gynaecological Endoscopy (SASREG) provides guidelines of standards to assisted fertility treatment for clinical services, nursing services, laboratory services and counselling. The guidelines are provided to the functional departments of public and private organisations for clinic self-assessment, and they submit their reports to SASREG for 4-year accreditation.

## Methods

The data on which we draw were collected as part of a large ethnographic study on travel for assisted reproduction and ova donation in Southern Africa. In total, we interviewed 117 informants (including patients, nurses and donors) from January 2022 to February 2023. This included key informants from across SSA (mainly South Africa, but including Uganda, Mozambique, Namibia, Tanzania, Ethiopia, Cameroon, Zambia and Ghana) and observations during visits to three public and six private clinics in Pretoria, Johannesburg, Mbombela and Cape Town (in September and October 2022). We supplemented this data with informal discussions with specialists, participation in professional meetings, and our observations of two fertility shows (2020 and 2022).

Below, we consider the themes that emerged primarily in interviews conducted with 19 fertility specialists, 11 embryologists, one clinic manager and three fellows registered in training programmes in South Africa. Most of our interlocutors were working in South Africa, but also elsewhere in southern and east Africa. Participants were recruited directly by personal introduction and through fertility clinics and personnel. Twenty-seven fertility specialists and embryologists were approached to participate; 19 agreed to be interviewed, two declined and six did not respond to our requests. Thirteen embryologists were approached for an interview, of whom 11 agreed, one declined and one did not respond. All interviewees were provided with information statements of the research project and provided formal, informed consent to be interviewed.

Interviews were conducted via Zoom and in person, the latter in their own offices and clinics. Recorded interviews were transcribed, and notes were taken and later thematically coded, then compared across the sample to note similar and contrasting opinions. Data collection was covered by the medical research ethics of two of the universities involved (see ethics statement), with data anonymised and stored on university servers in password-protected files. We have sought to protect the identities of our interviewees by using gender-neutral pronouns (they, their) and not to indicate race, although because the community of specialists is very small, there are limits to which we can ensure anonymity. In presenting the data, we have often quoted interviewees at length to capture the passion with which our interviewees spoke of the need to provide comprehensive reproductive health care and training to deliver this, underpinning our commitment, in turn, to reflect on their views.

## Results

### Current training options for fertility specialists

At the time of our research, training was available for fertility specialists in three public (teaching) hospitals in South Africa, the same hospitals that instituted fertility care and provided training when it was first available. Training in reproductive medicine takes 2 years full-time, following an undergraduate degree in medicine and surgery and subsequent clinical training. Gynaecologists can undertake study for the Sub-speciality Certificate in Reproductive Medicine of the College of Obstetricians and Gynaecologists of South Africa, with 2 years of training required in a numbered subspecialty trainee post. The present generation of fertility specialists are largely pioneers in the field in South Africa. Many trained overseas or were trained under a different system, primarily through rotation, until it was replaced by the system which applies today. One fertility specialist explained that during their period as a registrar in the public system, they rotated for about 2 months in the fertility unit and persisted in trying to get a post to the unit:

I remember at some point I thought I had gotten in but then someone resigned and then I was moved into uro-gynaecology and my professor just said it is all about the needs of the unit and not about our needs and we must consider that … When I started doing reproductive medicine there wasn’t a sub- speciality in South Africa … you just rotated ... the Department of Health needed to train us so that they could open units to start specializing in fertility treatments … So, we got in (under) what is called the ‘Grandfather clause’ … you have worked in fertility … (and) you need people to train the others in order to have units. So, I went through this process. They wanted logbooks, but there wasn’t an exam as such – but there is now, it’s [been in existence] two years now. It was one year [then], but now it is two, and you write an exam and … do research and … submit a thesis.

One specialist reflected: ‘our training system is quite rigorous… in other African countries you see that people go abroad for a couple of months, both biologists and clinicians, and then they are allowed to work … in the system’. Another fertility specialist estimated that now ‘it would take nearly 17 years to end up being a reproductive medicine specialist’ after they had completed initial training, internship, pre-residency, and residency, then two years to train in reproductive medicine. While this ensured good training, this specialist expressed concern that the time involved discouraged clinicians entering the specialisation, and on reflection, they felt that it wasn’t worth going into the field. However, as the first generation of fertility specialists is aging and nearing retirement, the lack of new cohorts foreshadows continuing difficulties in delivering ART; the same specialist said: ‘Yes, I don’t know what is going to happen when we go’.

Interviewees reiterated that demand outstripped supply; there were uneven resources in academic training units; and ‘the industry (private clinics) absorbs trained personnel without ploughing resources back to support the training institutions’. Training places in reproductive medicine are limited and reliant on partnerships between public and private institutions. At one teaching hospital, there was a 5-year waiting list for two full-time and two part-time training fellowships, but the numbers of specialists able to graduate are too few to meet training needs and demands from South Africa and elsewhere on the continent. There is too a risk that the delay might discourage some people from pursuing training, given the personal and direct costs of doing so. As one fellow reflected, potential trainees needed to balance their interest in the field, the value after training to them personally (in terms of income and personal commitments), the value to their clinic if the clinic paid for training, or to the hospital or country when their training was subvented to build local specialist capacity. As one committed fellow who was self-funded reflected, ‘it is a long process because it is four years (part-time) that you have got to do, and four years of your life when you basically have a quarter of your income, because it is not just losing out on income; you are actually spending money rather than making income’. Another fertility specialist noted that their private clinic paid for a fellow to come and study with them ‘because there weren’t enough teaching posts, so they came and trained with us through collaboration with (the university). But they weren’t paying them, we were paying them... They did four years with us as a fellow, and then wrote the exams, and then we invited them. They worked a year and then we invited them into partnership’. The length of time for training and financial imposition also works against greater diversity in the profession. Today, as historically, most fertility specialists in South Africa are white men.

### Challenges in training embryologists and clinical technologists

There is a shortage of embryologists as well as fertility specialist clinicians across SSA, and few can afford (or have a subvention or scholarship) to come to South Africa to train. At the time of interviewing, few people were training; within public institutions seven new biological scientists were being trained in reproductive biology at one public hospital and two at a second.

Public hospital services experience a constant drain of trained embryologists to the private sector. Those trained through the clinical technologist pathway, who complete training with privately owned authorised laboratories, are encouraged to and tend to stay within the private sector at those laboratories. Medical embryologists, trained through public sector academic institutions and laboratories (which also offer research training), often end up in the private sector, lured by better pay, access to high-end equipment and laboratories, opportunities for overseas training, and opportunities for extensive clinical experience due to the high clinical loads. While public institutions provide opportunities for further postgraduate degrees, limited subsidised places are available to allow those scientists to go on to further study. One drug company active in the region had established an annual public service donation to SASREG to promote education, training and research in public service academic laboratories, divided among three public registered training locations: Groote Schuur Hospital, University of Cape Town; Tygerberg Hospital, Stellenbosch University; and Steve Biko Hospital, University of Pretoria.

Our informants noted that approximately 15 people apply annually in South Africa to be trained in medical embryology; of these. only three are accepted due to limited training capacity. Trainees in medical embryology are carefully selected because, it was explained, embryologists must be able to carefully handle the ‘precious’ materials they are going to work with, and not everyone has this capacity. In their assessment for possible acceptance into the programmes, applicants spend a day in a lab to watch the work involved. The embryologists and medical scientists will score them on several qualities before they are invited for interview. At the interview, their motivation for training and their interest in the work is an important topic. As one interviewee explained, embryology was a scarce-skills profession in South Africa and they were committed to encouraging ‘independent evidence-based scientific thinking and life competencies’ as well as a strong sense of self-worth and professional capability.

Once trained, most embryologists are in high demand and may be lost from the public health system to work in the private sector. Several experienced embryologists in our sample had emigrated overseas, seeking further training opportunities and experience but also in some cases to live and work there permanently. As a result, across SSA, clinics complained about the difficulties in attracting and retaining embryologists and medical science staff.

### Motivations to study REI-ART

#### Neglect and the need for comprehensive care

Recognition of the relationship between the neglect of women’s sexual and reproductive health and their infertility motivated some fellows to commence training as fertility specialists because they saw the need to assist with conception and pregnancy and because they saw assisted reproduction as part of a continuum of care in the context of lack of treatment of reproductive tract medical problems and infections. Across the region, apart from age-related infertility and environmental exposures, infertility could be reduced by improved access to quality reproductive health care; including safe delivery and safe abortion care; contraceptive options to prevent unintended pregnancy; and the prevention, detection and treatment of infections. However, fallopian tube infections and consequent occlusion, largely due to STIs, post-partum infections, infections after pregnancy loss (primarily due to unsafe abortion) or tuberculosis, are often only detected when women want to become pregnant and can only conceive via access to ARTs (Ombelet 2011). At the same time, it has been estimated that up to 40% of difficulties in conception are due to male infertility (primarily teratozoospermia, see Akang *et al.* (2023)). Diagnosis of men’s infertility tends to be delayed because of stigma and assumptions that infertility is a woman’s problem (Dyer *et al.* 2004). This is in the context of the continued high burden of HIV in southern Africa: adult prevalence in Botswana, Eswatini, Zimbabwe and Lesotho is still over 20%, and South Africa has the highest number globally of people afflicted with HIV. Our interviewees located patients’ reproductive medical histories within broader histories of neglect, economic disadvantage and lack of treatment for reproductive medical problems, as one trainee explained:

The lack of financial wherewithal, lack of education, the lack of exposure to present to clinic or hospitals early. So a lot of people stay at home for all manner of reasons, and because of that an STI (sexually transmitted infection) develops, it propagates, it eventually causes a blocked tube.

One fellow contextualised this in relation to South Africa’s long HIV epidemic:

What I discovered was most of the patients [at the fertility clinic] were HIV positive and then when you are taking history you find that the patient previously had an STI. So obviously with my little knowledge at that time, but with those patients we would do like an HSG (hysterosalpingography) and you can see there is a blockage in the tube and there was no one to operate on these patients… and most of the time the women will be blaming themselves, right?

Delays to treatment exacerbate the development of further age-related infertility problems and infertility related to other gynaecological problems, as one trainee reflected:

The patient had a baby with her husband and now she comes back and doesn’t fall pregnant, and you do the investigation and ‘oh no, now she has grown fibroids’ or she has developed adenomyosis. Or you know, something has happened now, or her endometriosis has worsened and that is the reason why she can’t fall pregnant. So sometimes there are disease processes going on as you get older, that definitely affect your fertility ability, and I think this is what we see between the different age groups.

Comprehensive care requires that the full range of care is provided with the understanding that failure to intervene early increases the likelihood of more invasive procedures. One gynaecologist who was a trainee fellow in reproductive medicine, for example, became interested in assisted reproductive procedures from their interest in laparoscopic surgery. As they saw it, tubal ligation reversal, surgery for endometriosis, non-surgical cannulation or laparoscopy for blocked fallopian tubes and other fertility sparing procedures were all important interventions to assist women with conception and successful pregnancy, and IVF was the next (not the first) step in the event of continued failure to conceive. Assisted reproduction, including IVF and the use of donor gametes, was a critical point in a continuum of care which interviewees proposed; these measures were followed in turn by surrogacy (where legal) and, in their minds as the ‘last resort’, adoption. In their view, reproduction involving the gametes of both parents was ideal, with strong preference for intervention before the most invasive and technically complicated methods of assisted reproduction.

For another fellow, comprehensive reproductive health care ethically required that assisted reproduction be made available to intending parents following diagnosis of primary or secondary infertility and laparoscopic surgery to redress the effects of infection or disease. Their clinic lacked IVF capacity, however, and therefore they sought training: ‘A country cannot provide ART without that arm as well’. A fellow from outside of South Africa reflected that once a clinic or hospital was known to have qualified specialists, then people from ‘around the country and overseas’ would seek appointments there.

#### Gender and reproductive health care

Fellows linked their interest in ART to both their general interest in women’s health and to the specific gendered dimensions of infertility and subfertility: the pressure placed on women to reproduce; the stigma and suffering of women unable to conceive or successfully give birth; and the assumption that failure to conceive and reproduction was a woman’s responsibility. One fellow expanded on this:

And they come alone, you know? So even when we do the investigations we just focus on the female, and there is no male. … maybe a decade ago, I don’t even recall seeing a couple; you would see the female, and at that time you have no idea about the male factor … and yet, when you are dealing with infertility it is a couple, so you need to evaluate both male and female, and they were like, ‘no, but my husband has got three kids.’ You know? And you don’t even know if those kids are actually his. But there was never any sort of investigation, and you know, females are blaming themselves. And then you find out they are also struggling as well with the families; the mother-in-law now wants the son to get somebody else because this one is infertile.

As this suggests, interviewees mentioned that in certain cases, men were encouraged to take a second wife or to leave the first in order to have children. Women meanwhile were reluctant to bring their husbands into the clinic, perhaps, one fellow reflected, because of men’s power and status as breadwinners and household heads and the assumption that difficulties in conceiving were women’s fault and responsibility.

Interviewees noted that inappropriate care or failure to provide care to women for other sexual and reproductive health problems impacted on their fertility. Period pains due to endometriosis, for example, were assumed to be a sign of infection rather than dysfunction, and so women experiencing severe menstrual pain were treated regularly with antibiotics. Where a woman did have a sexually transmissible infection (STI), the partner was rarely also treated, so she would be reinfected:

Because remember with an STI, if you are treating a patient with an STI you have got to treat the partner. You are going to treat that patient and she is going to go back and sleep with the partner who could maybe give her the infection or whatever it is. And then this patient is going to be reinfected … You hardly see males with their females, it is always the females … And they are sort of blaming themselves, the females, and they become scared to actually discuss the condition that they have - even chlamydia. I think it is very important if you are treating the female, you have got to treat the partner!

Few gynaecologists and reproductive specialists in South Africa are women, and women specialists and fellows drew attention to the need for empathetic clinicians. In some cases this resulted in referral to a fertility specialist. Furthermore, clinicians as well as patients had limited knowledge of the factors that might contribute to infertility:

Even like qualified gynaecologists … they don’t tag infertility as a disease because it is unlike oncology where the patient is dying, they are losing weight and all sorts of things – and our patients are actually suffering psychologically and they actually need more time you know – even doing a consultation and listening to them. I can actually make a change in these patients, especially the couples that are suffering from infertility because it is a big issue … on the inside they are suffering, they are miserable.

### Professional socialisation

Fellows and reproductive specialists are not public health professionals but they acknowledged the need for counselling around reproduction, for public health interventions, and advocacy for improved diagnosis and treatment of reproductive medical problems and infections. Interviewees emphasised that health seeking was largely based on available finances and that people with low incomes often could not afford to seek care. But, they reiterated, their concerns were the ‘patient before them’ and the pressure on them from both the couple and family members: ‘So when they do come, we try and fix it. That is why I am saying to go out beyond the clinic and try to fix these issues is a big task’.

Key motivating factors to embark on specialist training as a reproductive specialist combined concern with the perceived need for care by women (especially) and the absolute lack of fertility specialists in Africa. However, fellows were also excited by new technical skills which would inform their practice (and so offerings of the clinic in which they worked and sometimes owned), by the quality of training and opportunities to build professional networks across the continent and beyond. As one interviewee noted, few professionals have opportunities for international mobility and access to resources. Fellows valued being able to attend conferences internationally when possible since it provided them with opportunities to meet suppliers and distributors of equipment and materials. These factors influenced people who trained decades ago – both embryologists and fertility specialists – and continue to inspire a current generation of fellows.

It also positioned fellows to reflect on the kinds of care they were able to offer women experiencing problems with fertility and the lack of care to prevent such problems. Within South Africa, there is considerable debate among fertility specialists over how best to reduce the costs of ARTs (Whittaker *et al.* 2024). The use of mild stimulation protocols and simplified laboratory procedures may reduce the cost of ART (Paulson *et al.* 2016, Nargund & Fauser 2020), but most private clinics in South Africa do not practice soft stimulation or ‘low-cost’ IVF. To date, the Walking Egg Program (WEP), which aims to provide simplified and cheaper laboratory embryo culture, remains experimental (Ombelet & Goossens 2016, Ombelet *et al.* 2025). Fellows knew little or nothing about low-cost ART until they began their fellowships, but in interviews they were eager to engage with debates about costs versus likely success and to elaborate on cost, efficiency and parsimony:

Low cost does not equate to low efficiency. So I think that is simply how I put it to patients. If you did high cost, I think probably the only thing that would change – and there is not a lot of evidence to even back it – but you are using normal dose stimulation and obviously you would generate more oocytes, that would obviously increase or improve your chances of fertilization plus duration and obviously live birth cases. But when you do high cost, you are doing genetic testing, but even genetic testing is not indicated in all situations. So there are lots of thrills and spills around the IVF that make it expensive but not necessarily efficient. So you can still get a good success rate doing the low cost approach.

And again:

I think that low cost is an important thing … unfortunately not everybody earns the same and I think that, you know, there are cases where women don’t necessarily need that much to stimulate – that you can actually do with much less, do a proper stimulation. … And at the end of the day the patient wants a baby in her arms – that’s what she wants; she doesn’t want to hear about cumulative pregnancy rate or … she wants to know about live birth rate, that is what she wants to know … you can do it in a way that is less expensive yet with the desired outcome.

Linked to this, fellows reflected on the (un)affordability of assisted reproduction treatment and the need for options to be provided, although one insisted that he simply gave patients ‘options’ from which to choose: ‘So I don’t stratify any of the patients based on economic circumstance, because I really don’t see it. I just see them as once they are here, let’s treat them, and let’s go’. Interviewees were mindful of the financial implications of infertility treatment: ‘If you have the money, then you are part of that’.

Against such circumspection, fellows, embryologists and fertility specialists were enthusiastic about the technology and the challenges of shaping the field: establishing a new clinic to meet previous unmet needs, for example, identifying suppliers for laboratory and clinic consumables (liquid nitrogen and culture media), ensuring the service of instruments, training managers and the like. Allied to training and shared discussion about current and desired practices of care, interviewees were also adamant of the need for embryologists. This was because of their key task as a trained scientist but also, in the context of the use of donor gametes, because the embryologist undertook the counselling – ‘the cheapest route is actually to get donor sperm or donor gametes, obviously it is just a lot of counselling and patient education that you need and things like that’. The distress of patients in relation to their infertility escalates as specialists seek to hold down costs: ‘Because they have been trying to fall pregnant, most of them are frustrated, they can feel very aggressive, they are agitated, and now you have to convince them that you only want to stimulate them for one or two oocytes. They want to try and get as much as they can’.

### Providing services in the future

Present day fertility specialists noted the recent or imminent retirement of many of their colleagues in current public and private practice. Yet the present training system is not seen as producing enough specialists to replace them, let alone expand services to meet future demands in SSA. Setting up and running ART clinics in SSA is therefore limited. Clinic staff from Ghana, Uganda and Kenya who we interviewed described their need to employ a constant stream of expatriate embryologists to work in their laboratories, of whom few stayed for long periods of time. Some fertility specialists and embryologists are what was called by one embryologist ‘moving and roving’ or FIFO (fly in, fly out) staff travelling in circuits to associated clinics in other countries for regular short periods of time. This is generally unsustainable and requires the ‘batching’ of cohorts of patients (a controversial practice that may impinge upon quality of care) and limits available services (ref removed for anonymity). Likewise, efforts to introduce low-cost IVF schemes are limited by the human resources still required. As specialists and embryologists reflected, years of clinical practice after initial training is needed to be skilled and experienced, and so to trouble-shoot problems; they were sceptical of schemes to train embryologists at speed – in 3 months in India, for instance – to expand low-cost services. All recognised continued issues with low pay, lack of status and recognition, and career progression for embryologists, despite their crucial roles in ART. We interviewed experienced embryologists from SSA who had moved overseas to Australia or the United States, where they could command higher wages and secure opportunities for their families unavailable in their home countries. Similarly, a few fertility specialists expressed concern that they had found the need to move from the public sector into private practice to secure the economic future of their families and to gain greater clinical experience than was possible with the fewer women being treated in public clinics.

## Discussion and conclusion

Limited access to quality fertility care and sufficient trained clinicians and other specialists are not unique to SSA. Worldwide there is growing demand for REI-ART specialists with the expansion of assisted reproduction and the emerging middle classes who can afford these services (de Ziegler *et al.* 2015*b*). Reproductive gynaecologists and other specialists regularly draw attention to the need to expand local high-quality training in REI-ART in lower- and middle-income countries (LMIC) where the problem is most acute; even within the US there is geographic maldistribution in the opportunities for clinics, physicians and training programs (Diego *et al.* 2023). Similarly, for embryologists a review revealed significant international differences in embryologist certification and duties across countries, which will impact future staffing (Shirasawa & Terada 2025).

Different models of training have been proposed to address this shortfall, such as linking a reference training centre to satellite practical training sites, and REI-ART specialists have advocated combining this with scenario simulation training models to achieve competency before initiating live clinical experience. The use of virtual and online training materials is recommended as a means of fulfilling training needs worldwide (de Ziegler *et al.* 2015*a*). Our study suggests that in SSA a survey of workforce training needs and a curriculum review are necessary to best plan for future workforce shortages in the sector; to determine whether current training fits present needs, and to identify whether other modes of delivery or shorter intensive training and scaffolded professional development might assist shortfalls. Virtual delivery of content already happens to a limited extent through the European Society for Human Reproduction and Embryology workshops and online regional seminars. Collaborative standardised international training schemes across professional organisations, associations and institutions to support exchanges for medical staff for ongoing training and support of academic training institutions in SSA could be expanded, but this would require careful pedagogical and context-specific design and institutions committed to long-term relationships.

Clinical experience is crucial in REI/ART training, and schemes are needed to address the shortage of placements and overcome the significant financial barriers. The introduction of more automation within laboratories may result in opportunities to reconsider workflows, and this suggests potential in reorganising the roles of highly skilled staff with those with lower qualifications to handle more routine tasks (Shirasawa & Terada 2025).

South Africa is the largest hub for assisted reproduction and training of REI-ART staff for SSA. The shortages in skilled fertility specialists and embryologists contribute to the inequitable access to assisted reproduction across the continent. In our study, fertility specialists and trainee fellows emphasised the need for gynaecologists to improve their fertility knowledge to provide men and women with appropriate andrology and gynaecological advice and treatment, with assisted reproduction presented as a resort only once other options had been explored. Such primary fertility care would reduce the burden upon fertility specialists. Nevertheless, as described above, there are limited available opportunities for clinicians wishing to specialise in fertility and assisted reproduction to receive specialist training in South Africa or elsewhere, and this may come with significant personal, financial and time investment costs. Waiting times for admission to these programmes can be years. Still, trainee fellows who enter the training programmes are highly motivated. While a greater number of training opportunities exist for embryologists, again there is a dire shortage across SSA. In general, policies, programmes and spokespeople regard infertility care and assisted reproduction of lesser importance to other reproductive and health problems; in doing so, these underestimate the extent and impact of infertility, the demand for ART, and the failure to meet people’s reproductive and sexual health needs and rights.

Trainee fellows are committed to low-cost IVF models as one of several options for appropriate reproductive health care. Ongoing research towards a pilot programme with this in mind (Boshoff *et al.* 2018) provides some optimism that this is possible, although there is scepticism that the approach will provide opportunities to conceive (removed for anonymity). But even if this were to lead to an expanded and affordable programme, it would only be possible with trained embryologists, medical biologists and fertility specialists. In consequence, intending parents with ART will continue to struggle for care unless training needs are given priority.

## Declaration of interest

The authors declare that there is no conflict of interest that could be perceived as prejudicing the impartiality of the work reported.

## Funding

This research was supported by the Australian Government through an Australian Research Council Discovery Project Grant (DP 200101270).

## Author contribution statement

All authors contributed to the design and implementation of the research, the analysis of the results, and the writing of the manuscript.

## Data availability

The datasets used and analysed in the current study are not available to be shared openly due to conditions specified in our ethics approvals to protect participant confidentiality given the small number of specialists in SSA.

## Ethics approval and consent to participate

Ethics clearance was granted by Monash University (MUHREC 27166), the University of the Witwatersrand (HREC (Medical) M210546) and participating clinics.
